# Keratin 17 Is Required for Lipid Metabolism in Keratinocytes and Benefits Epidermal Permeability Barrier Homeostasis

**DOI:** 10.3389/fcell.2021.779257

**Published:** 2022-01-12

**Authors:** Bingyu Pang, Zhenlai Zhu, Chunying Xiao, Yixin Luo, Hui Fang, Yaxing Bai, Zhongbin Sun, Jingyi Ma, Erle Dang, Gang Wang

**Affiliations:** Department of Dermatology, Xijing Hospital, Fourth Military Medical University, Xi’an, China

**Keywords:** epidermal barrier, Keratin 17, lipid metabolism, fatty acid synthase, sterol regulatory element-binding protein 1, peroxisome proliferator-activated receptor gamma

## Abstract

The epidermal barrier refers to the stratum corneum, the uppermost layer of the skin, and constitutes the first line of defense against invasion by potentially harmful pathogens, diminishes *trans*-epidermal water loss, and plays a crucial role in the maintenance of skin homeostasis. Keratin 17 (K17) is a type I epithelial keratin with multiple functions, including in skin inflammation, epithelial cell growth, protein synthesis, and tumorigenesis. However, the relationship between K17 and the skin barrier has yet to be systematically investigated. In this study, we found that acute disruption of the epidermal permeability barrier led to a rapid increase in epidermal K17 expression *in vivo*. *Krt17* gene deficiency in mice resulted in decreased expression of lipid metabolism-related enzymes and antimicrobial peptides, while also delaying epidermal permeability barrier recovery after acute disruption. Adenovirus-mediated overexpression of K17 enhanced, whereas siRNA-mediated knockdown of *Krt17* inhibited, the expression of fatty acid synthase (FASN) and that of the transcription factors SREBP-1 and PPARγ *in vitro*. We further confirmed that K17 can facilitate the nuclear transportation of SREBP-1 and PPARγ and promote lipid synthesis in keratinocytes. This study demonstrated that K17 contributes to the restoration of the epidermal permeability barrier *via* stabilizing lipid metabolism in keratinocytes.

## Introduction

The epidermal barrier is primarily constituted by the stratum corneum (SC), which is the outer layer of the skin, and represents a robust barrier against external environmental stressors as well as a water-tight barrier that prevents *trans*-epidermal water loss (TEWL). The loss of structural and biophysical homeostasis can provoke or aggravate chronic skin disorders (Egawa and Kabashima, 2016; Yosipovitch et al., 2019). The epidermal barrier is comprised of protein-enriched corneocytes embedded in an intercellular lipid matrix, called the “brick and mortar” model. The cellular complement is immersed in an intercellular lipid “mortar” composed of ceramides, cholesterol, and free fatty acids (FFAs) (Nemes and Steinert, 1999; Jia et al., 2018). Aberrant key steps in lipid metabolism in keratinocytes can result in alterations to barrier lipid components and thereby weaken epidermal barrier functionality (Akiyama, 2017). However, the precise mechanisms underlying the regulation of lipid metabolism in keratinocytes and the maintenance of skin homeostasis remain poorly understood.

Keratin 17 (K17), a type I epithelial keratin intermediate filament protein, is widely distributed in basal cells of complex epithelia, including hair follicles, sebaceous glands, fingernails, and eccrine sweat glands (Kurokawa et al., 2011). Physiologically, K17 plays a key role in maintaining normal hair follicle functions; accordingly, mice deficient for *Krt17* exhibit severe alopecia after birth resulting from TNF receptor-mediated apoptosis (McGowan et al., 2002). Although K17 is undetectable in normal *epidermis*, it is highly expressed in some skin disorders, in which it accelerates keratinocyte proliferation and promotes inflammation (Jiang et al., 2017; Yang et al., 2017; Yang et al., 2018). Moreover, *Krt17*-null mouse embryos show delayed wound closure due to decreased AKT/mTOR signaling activity, thereby revealing a critical role for K17 in skin repair (Kim et al., 2006). Nevertheless, whether and how K17 contributes to epidermal barrier function remains unknown.

Recently, our group noticed that K17 expression was upregulated in the *epidermis* after barrier perturbation, and altered lipid metabolism in keratinocytes K17. This suggested that K17 plays a critical role in epidermal barrier repair. In this study, we assessed the role of K17 in epidermal barrier function using *Krt17* knockout mice and then validated the findings in HaCaT cells. The results showed that K17 expression was upregulated following acute disruption of the epidermal barrier, an effect that promoted the recovery of the skin barrier *via* the modulation of lipid metabolism. These findings offer a novel viewpoint on the biological role of K17 and suggest a new therapeutic strategy for regulating epidermal barrier function.

## Materials and Methods

### Mice and Treatment

*Krt17* knockout mice in a C57BL/6J background were kindly provided by Prof. Pierre A. Coulombe (Johns Hopkins University, Baltimore, MD, United States). Female mice, 6–8 weeks old, were used in the experiments. All experimental procedures were performed in compliance with the National Institutes of Health Guide for the Care and Use of Laboratory Animals and approved by the Review Committee for the Use of Animals of the Fourth Military Medical University. In the acute barrier abrogation model, barrier permeability was disrupted in mice by repeated applications of cellophane tape on the shaved back until a 10-fold increase in TEWL levels was achieved (Tsai et al., 1994). SC hydration and TEWL were measured immediately (0 h) and at 3 and 6 h after barrier disruption using a multifunctional skin physiology monitor (MPA10, Courage-Khazaka Electronic GmbH). The skin was collected 6 h after acute disruption, and the *epidermis* was either separated from the dermis by heat separation (Man et al., 2015) or fixed in formalin for histopathological analysis. The recovery rate was calculated as follows:

Recovery rate=TEWL (0 h)−TEWL (3 h or 6 h)TEWL (0 h)×100%

### Cell Culture and Transfection

Human HaCaT keratinocytes (American Type Culture Collection, Manassas, VA, United States) were cultured in Dulbecco’s modified Eagle’s medium (Gibco, Grand Island, NY, United States) supplemented with 10% fetal bovine serum (Gibco) and maintained at 37°C in a humidified atmosphere containing 5% CO_2_. HaCaT cells were transfected either with pEGFP-N1-K17 or short interfering RNA (siRNA) targeting K17 using Lipofectamine 3,000 (Invitrogen; California; United States). The siRNA sequences for K17 are listed in Supplementary Table S1.

### RNA extraction and Real-Time Quantitative PCR Analysis

Total RNA was extracted using Trizol reagent (Takara, Tokyo, Japan) and purified using chloroform/isopropanol/ethanol. The extracted RNA (1 µg/10 µl reaction) was converted to cDNA using the Prime Script RT Master Mix Kit (Takara). Quantitative real-time PCR (qPCR) was performed using SYBR Premix Ex Taq II (Takara) on a Chromo4 Continuous Fluorescence Detector with a PTC-200 DNA Engine cycler (Bio-Rad, CA; United States). The cycling conditions were as follows: 95°C for 2 min, followed by 45 cycles of denaturation at 95°C for 5 s, annealing at 60°C for 10 s, and extension at 72°C for 15 s. Relative quantification was performed using the ΔΔCT method. All reactions were run in triplicate for at least three independent experiments. Data are expressed as a percentage of control (setting controls as 100%). The sequences of the primers used are listed in Supplementary Table S2.

### Immunofluorescence and Confocal Microscopy

Biopsies obtained from the skin of the back of the mice were fixed in 12% formaldehyde solution and embedded in paraffin. For immunofluorescence (IF) staining, cells or skin biopsy specimens were permeabilized with 0.5% Triton X-100 for 10–15 min at room temperature. After washing three times with PBS, the cells or skin biopsy specimens were incubated with primary antibodies targeting K17 (sc-393002; Santa Cruz Biotechnology), SREBP-1 (IgG-2A4; BD Biosciences), or PPARγ (81B8; Cell Signaling Technology) at 4°C overnight, washed three times with PBS, and then incubated with Cy3-, fluorescein isothiocyanate (FITC)- (for cells transfected with siRNA and tissue slices), or Alexa Fluor 647-conjugated (for cells transfected with pEGFP-N1-K17) secondary antibodies (ab6939, ab6785, and ab150075, respectively; Abcam); nuclei were counterstained with Hoechst 33,258 (Solarbio Technology; Beijing; China). The samples were observed and imaged using a confocal microscope (LSM880; Carl Zeiss; Germany).

### Western Blot Analysis

Western blot was performed as previously described (Jian et al., 2011) using the following antibodies: anti-K17 (ab53707; Abcam), anti-FASN (ab128856; Abcam), anti-SREBP-1 (PA1-337; Invitrogen), anti-PPARγ (81B8; Cell Signaling Technology), anti-β-tubulin (10068-1-AP; Proteintech), anti-β-actin (66009-1-Ig, Proteintech), anti-GAPDH (60004--1-Ig; Proteintech), and anti-lamin-A/C (sc-7292; Santa Cruz Biotechnology). Band intensities were quantified using Image Lab version 5.2.1 (Bio-Rad). Relative band intensities were normalized to that of the loading control.

### Co-Immunoprecipitation

After the respective experiments, treated cells were subjected to co-IP assays [anti-K17 (sc-393002; Santa Cruz Biotechnology) or anti-IgG (TA-02; Origene, MD, United States) antibodies] using Protein A/G PLUS-Agarose (sc-2003C; Santa Cruz Biotechnology) according to the instructions of the manufacturer. Whole-cell lysates were purified in lysis buffer and incubated with anti-K17 or anti-IgG antibody on a rocker platform for 3 h followed by incubation with Protein A/G PLUS-Agarose at 4°C overnight. After four PBS washes, the supernatant was discarded and the pellets were resuspended in 1× electrophoresis sample buffer for immunoblot analysis.

### Oil Red O Staining

Transfected HaCaT cells or frozen sections were fixed in 4% paraformaldehyde (PFA) for 10 min at room temperature and incubated in 0.5% Oil Red O (O8010-5; Solarbio) solution for 30 min. Tissue sections were then experienced a counter staining for nuclei with hematoxylin for 2 min. Images of cells were recorded using a FV-1000S confocal microscope (Olympus). The stained areas were detected and quantified (adjusted to cell quantity) using the ImageJ v1.8.0_172 (NIH, United States). Biopsies were scanned by NDP view system (Hamamatsu, Japan).

### Statistical Analysis

The data were analyzed using the unpaired, two-tailed Student’s *t*-test or one-way analysis of variance in GraphPad Prism v.8.0 (GraphPad Software, La Jolla, CA, United States). Each experiment was performed at least three times. Values of *p* < 0.05 were considered statistically significant.

## Results

### The Expression of Keratin 17 was Upregulated Following the Disruption of the Epidermal Permeability Barrier in Wild-Type Mice

**FIGURE 1:**
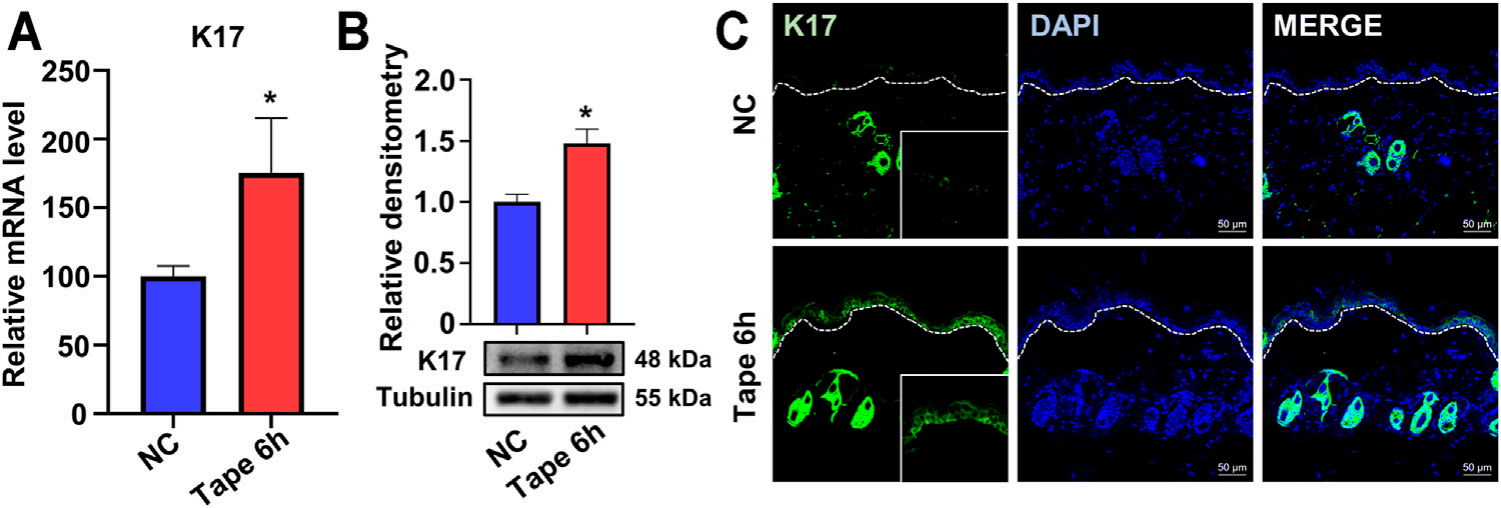
The expression of keratin 17 was upregulated after acute epidermal disruption in wild-type mice. **(A)** The mRNA levels of keratin 17 (*Krt17*) in the *epidermis* 6 h after tape stripping as detected by real-time quantitative PCR (RT-qPCR). **(B)** The protein levels of K17 as determined by Western blot; quantification was based on three independent experiments. **(C)** Immunofluorescence staining for K17 (green). *p<0.05.

To explore the changes in K17 expression after skin barrier disruption, acute disruption of the epidermal permeability barrier was instigated by repeated tape stripping on the backs of wild-type (WT) mice. Compared with control, undisrupted *epidermis*, *Krt17* mRNA levels were significantly upregulated 6 h after tape stripping as revealed by RT-qPCR (Figure 1A) and further confirmed by Western blotting (Figure 1B). Consistent with this, IF staining showed a marked accumulation of K17 in the *epidermis* of the test group; in contrast, K17 expression was barely detectable in normal interfollicular *epidermis* (Figure 1C). These results indicated that K17 could be induced following acute insult to the epidermal permeability barrier.

### The Lack of *Krt17* Delayed Epidermal Permeability Barrier Recovery

**FIGURE 2:**
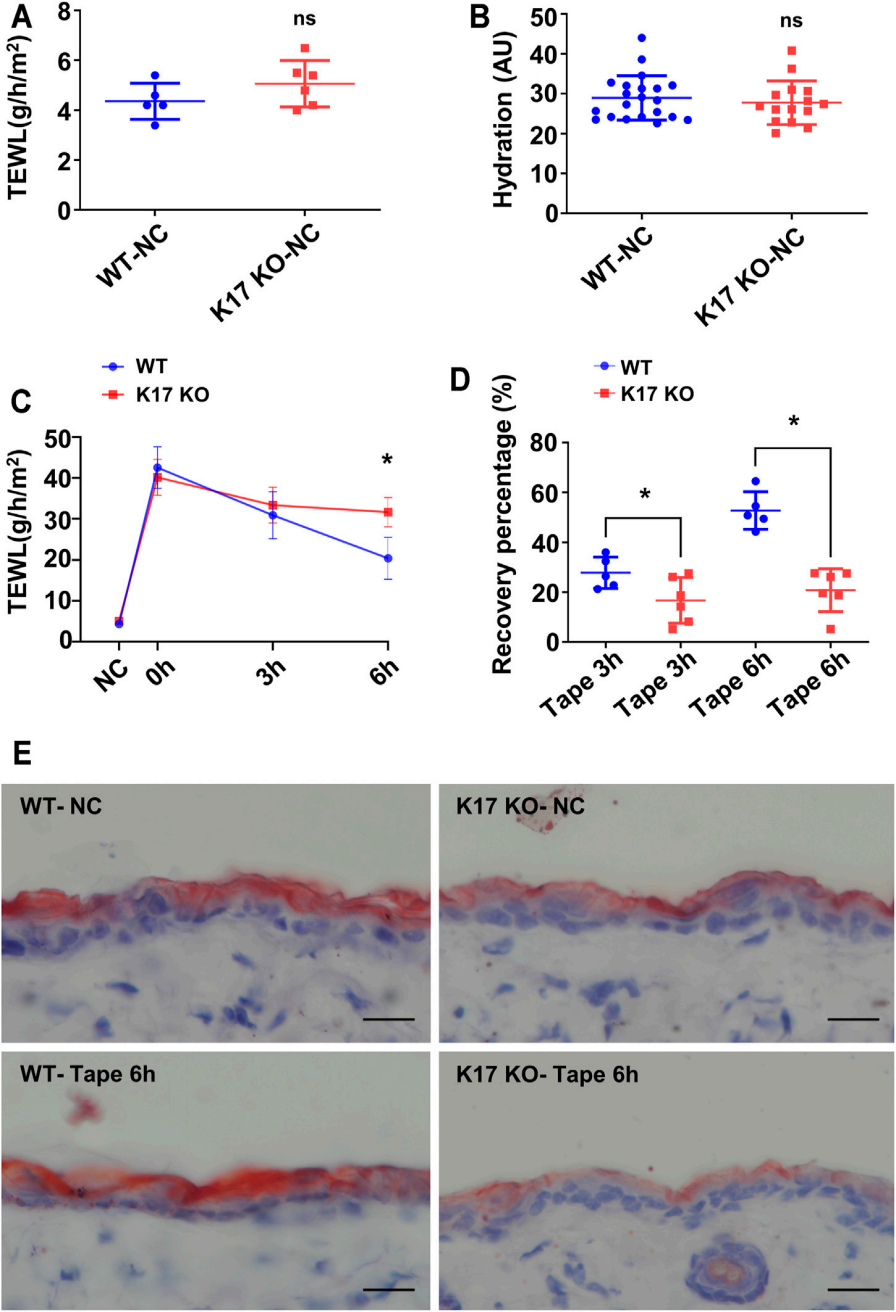
The ability of the epidermis to repair itself was impaired in keratin 17-null mice. **(A)** *Trans*-epidermal water loss (TEWL) in keratin 17 (*Krt17*) knockout and wild-type (control) mice before tape stripping. **(B)** The basal levels of epidermal hydration in *Krt17* knockout and wild-type (control) mice. **(C)** TEWL monitoring at 0, 3, and 6 h after permeability barrier disruption. **(D)** Recovery rate at 3 and 6 h. **(E)** Oil Red O staining of biopsies at steady state and 6 h after disruption. Scale bars = 20 μm*p < 0.05.

To clarify the effect of K17 on the epidermal permeability barrier, skin barrier function and recovery after injury were investigated in *Krt17* knockout mice. TEWL serves as a reliable readout of permeability barrier status *in vivo* (Hu et al., 2017). No differences in basal TEWL levels (Figure 2A) or SC hydration status (Figure 2B) were detected between WT and *Krt17* knockout mice. Subsequently, we compared the TEWL levels between the two groups of mice immediately (0 h after tape stripping) and 3 and 6 h after tape stripping-induced epidermal barrier disturbance. We found that the TEWL levels of WT mice showed a marked decline, whereas those of *Krt17*-null mice exhibited little change (Figure 2C). Additionally, compared with WT mice, the recovery rate of *Krt17* knockout mice was significantly delayed at 6 h after skin disruption (Figure 2D). Oil Red O staining was also performed to illustrate the lipid matrix. It was clearly demonstrated that lipid staining of *Krt17* knockout mice dorsal skin at steady state exhibited a moderate reduction compared with WT mice. Moreover, epidermal lipids were slightly increased 6 h after tape stripping in WT mice, whereas those of *K17* null mice had a declined intensity compared with that of untreated skin samples, which is probably due to the delayed lipid generation. The staining results illustrated an insufficient lipid production in *Krt17* knockout mice *epidermis* after barrier disruption (Figure 2E). Taken together, these results suggested that K17 is essential for skin barrier recovery rather than its maintenance at steady state.

### The Relative Expression Levels of Genes Encoding Lipid Synthesis-Related Enzymes Were Downregulated in the Epidermis of *Krt17* Knockout Mice

**FIGURE 3:**
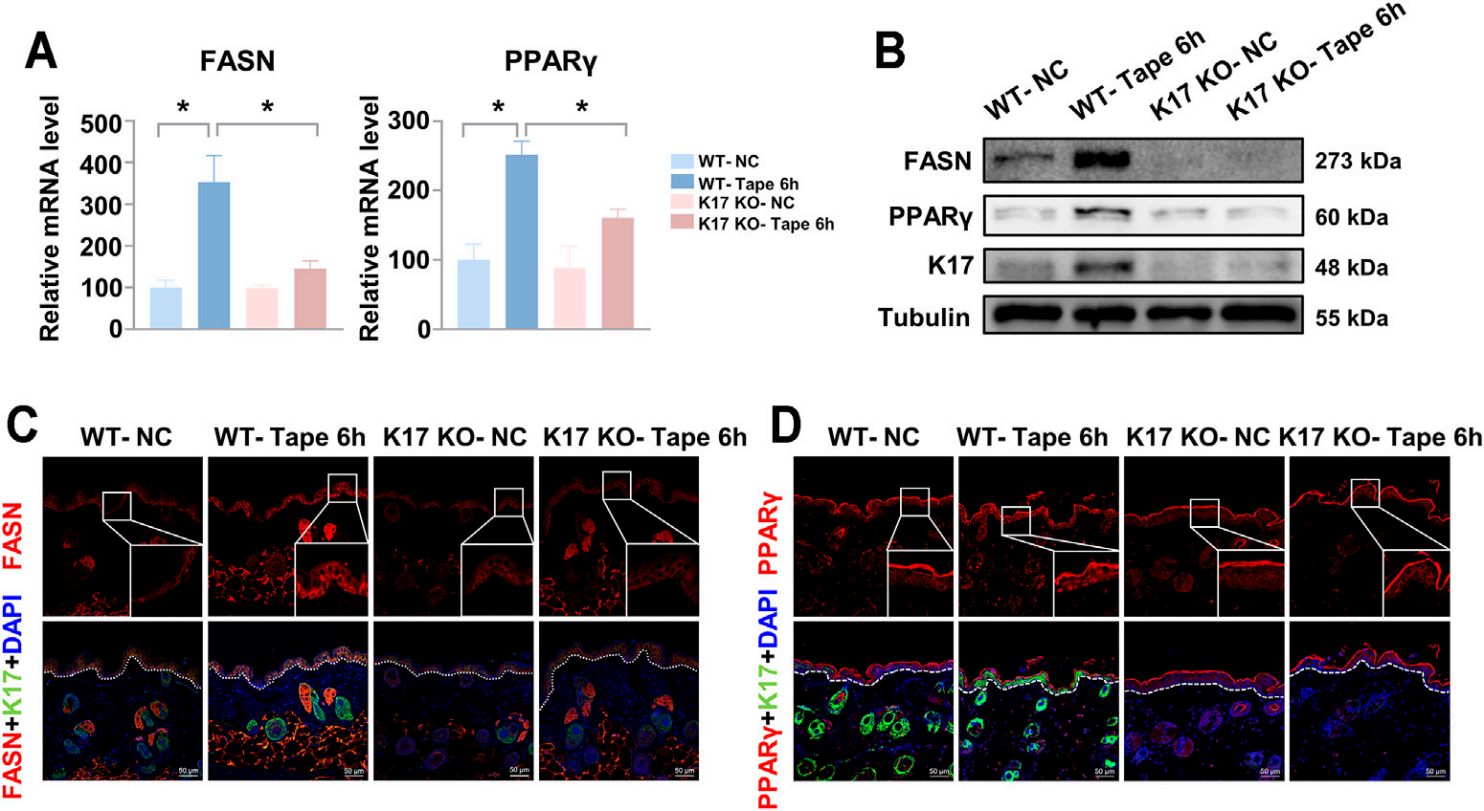
Keratin 17 knockout abolishes the upregulation of fatty acid synthase (FASN) (epidermal lipid synthesis enzyme) and peroxisome proliferator-activated receptor gamma (PPARγ) (transcription factor) expression after epidermal barrier disruption *in vivo*. The shaved skin in the backs of keratin 17 (*Krt17*) knockout and wild-type (control) mice was disrupted using tape stripping. The skin was collected for analysis 6 h after tape stripping. **(A)** Quantitative real-time PCR results of the relative mRNA expression levels of the epidermal lipid synthesis enzyme, FASN and the transcription factor, PPARγ. Data were normalized to non-tape-stripped wild-type controls (controls were set as 100%). Data are representative of at least three independent experiments and each group consisted of three mice. **(B)** The protein levels of FASN, PPARγ, and K17 were measured in each group of mice. Tubulin served as the loading control. **(C,D)** Immunofluorescence staining of FASN and PPARγ. Scale bars = 50 μm. Results are shown as means ± SEM. *p < 0.05.

To determine the effect of K17 on key genes associated with epidermal function, the relative expression levels of a plethora of genes related to epidermal barrier function, including proliferation, differentiation, lipid metabolism, and antimicrobial activities, among others (Mizutani et al., 2009; Svoboda et al., 2016; Zhong et al., 2016; Danso et al., 2017), were assessed by RT-qPCR. The results showed that the mRNA expression levels of genes associated with keratinocyte proliferation, differentiation, SC hydration, and tight junctions were comparable between *Krt17* knockout and WT mice (Supplementary Figures S1A–E). However, the expression levels of genes encoding the antimicrobial peptides S100A8, S100A9, and LL-37 were markedly reduced in *Krt17*-null mice compared with those in WT mice (Supplementary Figure S1F). Importantly, relative to the WT controls, *Krt17* knockout mice exhibited a significant reduction in fatty acid synthase (*Fasn*) and peroxisome proliferator-activated receptor gamma (*Pparg*) levels after tape stripping (Figure 3A). Western blot and immunofluorescent staining results were consistent with those obtained by qPCR analysis (Figures 3B–D). Combined, these findings demonstrated that *Krt17*-null mice display lower mRNA expression levels of genes encoding lipid metabolism meditators and antimicrobial peptides.

### Keratin 17 Regulates Fatty Acid Synthase and Peroxisome Proliferator-Activated Receptor Gamma Expression in Keratinocytes

**FIGURE 4:**
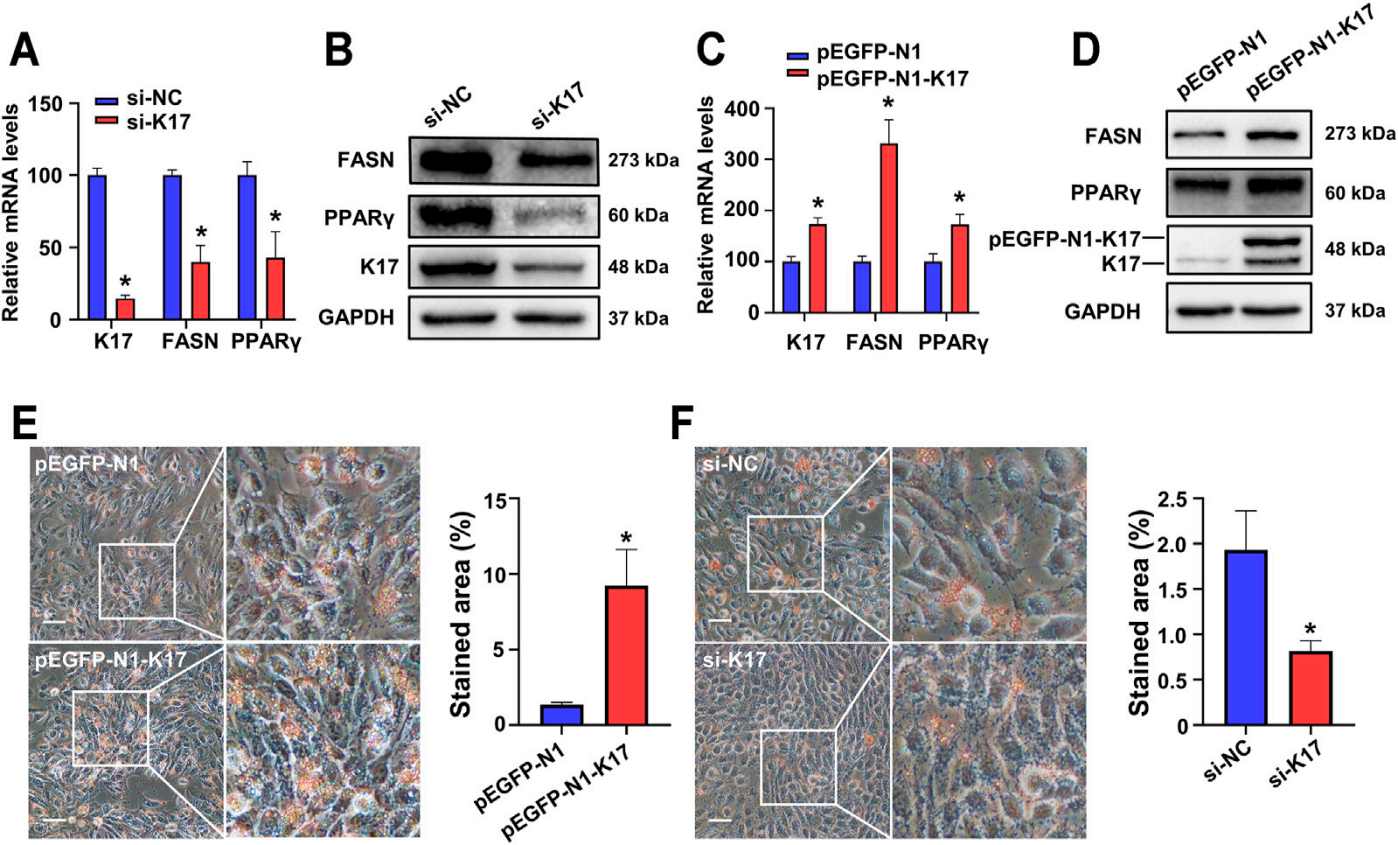
K17 in keratinocytes maintains and regulates FASN and PPARγ expression. **(A,B)** The mRNA and protein levels of K17, FASN, and PPARγ in the keratinocyte cell line HaCaT were measured after transfection with K17 siRNA. **(C,D)** The mRNA and protein levels of K17, FASN, and PPARγ in HaCaT cells were measured after transfection with pEGFP-N1-K17. Data are expressed as means ± standard error of the mean of three independent experiments. **(E,F)** Lipid droplets in HaCaT cells transfected with pEGFP-N1-K17 or K17 siRNA were stained with Oil Red O and visualized by light microscopy at ×400 magnification. Scale bars = 50 µm *p < 0.05.

To confirm the results obtained *in vivo*, HaCaT cells were transfected with siRNA targeting KRT17 (si-K17) following which the mRNA and protein expression of FASN and PPARγ was analyzed by RT-qPCR and Western blot, respectively. We found that the expression of both FASN and PPARγ was downregulated in K17-depleted cells at both the mRNA (Figure 4A) and protein (Figure 4B) levels. HaCaT cells were also transfected with a K17 overexpression plasmid (pEGFP-N1-K17). As shown in Figures 4C, D, the overexpression of K17 led to the induction of both the mRNA and protein expression of FASN and PPARγ. These findings indicated that K17 positively regulates FASN and PPARγ expression in keratinocytes. Subsequently, we sought to determine the overall influence of K17 on lipid metabolism using Oil Red O staining. The results clearly demonstrated that, compared with the controls, lipid staining area was greater in HaCaT cells transfected with pEGFP-N1-K17 (Figure 4E) and smaller in si-K17-transfected cells (Figure 4F). Taken together, these results suggest that K17 exerts positive effects on lipid metabolism *via* the modulation of FASN and PPARγ expression.

### Keratin 17 Promotes the Nuclear Localization of Sterol Regulatory Element-Binding Protein 1 and Peroxisome Proliferator-Activated Receptor Gamma, Thereby Inducing the Expression of Lipid Synthesis-Related Enzymes

**FIGURE 5:**
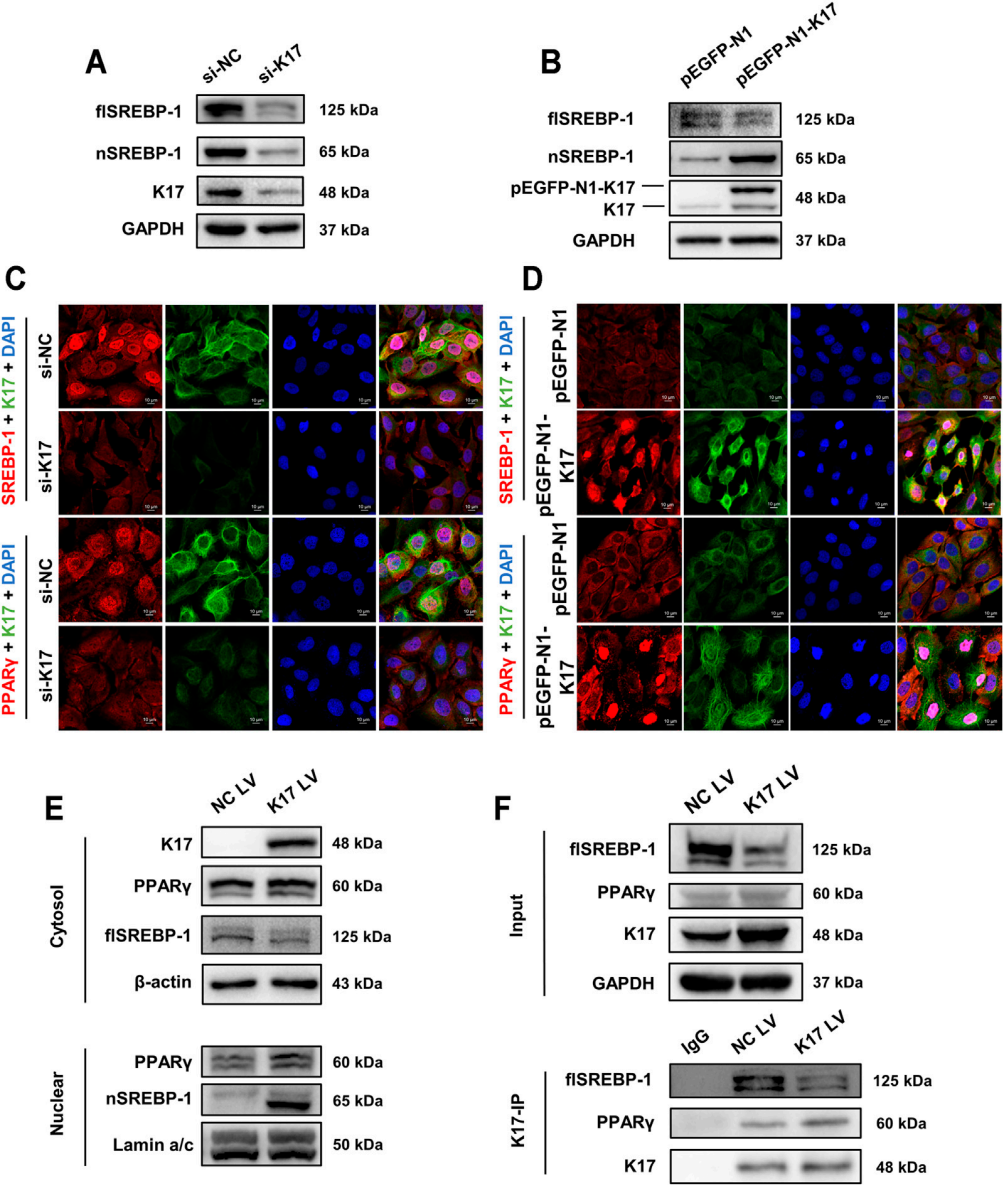
K17 alters the subcellular localization of SREBP-1 and PPARγ. **(A,B)** The protein levels of full-length SPEBP-1 (flSREBP-1) and nuclear SPEBP-1 (nSREBP-1) in HaCaT cells were measured after transfection with K17 siRNA or pEGFP-N1-K17. **(C,D)** Immunofluorescence analysis of FASN and PPARγ subcellular localization in HaCaT cells after transfection with K17 siRNA or pEGFP-N1-K17. Scale bars = 10 μm. **(E)** HaCaT cells were treated with pCMV6-XL5-K17 (K17 LV) and a nuclear fraction was prepared. SREBP-1 and PPARγ protein was quantitated by Western blot. Lamin A/C and β-actin were used as loading controls for nuclear and cytosol fractions, respectively. **(F)** Cells were co-immunoprecipitated with anti-K17 antibody and SREBP-1 and PPARγ proteins were detected by Western blot. Data are expressed as means ± standard error of the mean from three independent experiments. *p < 0.05.

Several studies have demonstrated that sterol regulatory element-binding protein 1 (SREBP-1) is a transcription factor for a series of lipid metabolism-related enzymes such as acetyl-CoA carboxylase (ACC), stearoyl-CoA desaturase-1 (SCD-1), and, notably, FASN (Shimano and Sato, 2017). Accordingly, we sought to determine whether a correlation exists between K17 expression and SREBP-1 and PPARγ protein levels. The Western blotting results showed that inhibiting K17 using siRNA resulted in the downregulation of full-length SREBP-1 (flSREBP-1) and nuclear SREBP-1 (nSREBP-1), whereas the overexpression of K17 led to a reduction in flSREBP-1 levels but an increase in the levels of cleaved SREBP-1 (Figures 5A, B). As shown in Figure 5C, nuclear staining for SREBP-1 and PPARγ was barely discernable following K17 knockdown, whereas the opposite was observed when K17 was overexpressed (Figure 5D), as determined by IF. These results were further confirmed by Western blot analysis of separated cytoplasmic and nuclear fractions obtained from treated cells (Figure 5E).

Co-immunoprecipitation using anti-K17 antibody was then performed to determine whether K17 can potentially bind to SREBP-1 and PPARγ (Figure 5F). The results suggested that K17 expression was positively correlated with nuclear-localized SREBP-1 and PPARγ and that K17 directly interacted with SREBP-1 and PPARγ and directed their nuclear translocation.

## Discussion

In the present study, we found that K17 expression was upregulated following tape stripping-induced skin barrier disruption. We further found that, in comparison with WT mice, *Krt17* knockout mice exhibited delayed barrier recovery and reduced FASN and PPARγ expression levels during barrier reconstruction. Additionally, our data showed that K17 influenced the subcellular localization of SREBP-1, a key transcription factor for FASN and PPARγ, leading to changes in lipid metabolism in keratinocytes.

K17 is only weakly expressed in normal human *epidermis*, but is inducible under conditions of stress, such as after wounding, viral infection, tumor growth, and skin diseases (Kim et al., 2006; Jin and Wang, 2014; Mikami et al., 2015; Yang et al., 2017). Our data further confirmed this, in that we found that epidermal barrier disruption, a less severe insult, is sufficient to induce the expression of K17. Multiple roles have been ascribed to K17 to date. The loss of K17 protein leads to a dose-dependent delay in the closure of embryonic skin wounds, highlighting the critical role of K17 in re-epithelialization during skin repair (Mazzalupo et al., 2003). Studies have shown that K17 can bind to adaptor protein 14-3-3δ and thereby facilitate keratinocyte proliferation and protein synthesis through phosphoinositide 3-kinase (PI3K)/protein kinase B (AKT)/mammalian target of rapamycin (mTOR) signaling (Kim et al., 2006). In addition to cell proliferation, high K17 expression levels have also been linked with inflammatory skin diseases, such as psoriasis and atopic dermatitis. We have previously documented the roles of K17 in attracting inflammatory cytokines, promoting T-cell infiltration, and the thickening of the *epidermis* in psoriasis, as well as the associated molecular mechanisms (Shi et al., 2011; Jin and Wang, 2014; Yang et al., 2017; Yang et al., 2018). In the present study, we found that acute barrier disruption resulted in increased expression of K17 at both the mRNA and protein levels and that K17 deficiency compromised barrier repair. Our data further revealed a novel function for K17 in modulating lipid metabolism in keratinocytes. The delayed wound healing observed in *Krt17*-null mice from an early stage suggests that the protective role of K17 in the maintenance of barrier homeostasis may involve at least two distinct mechanisms, namely, cell proliferation and lipid synthesis. These results establish K17 as an essential regulatory factor in restructuring irritated *epidermis*.

Extracellular lipids in the *epidermis* mainly refer to lipids of the SC, primarily including ceramides, FFAs, and cholesterol. Variations in the fatty acid profile have been found to correlate with skin disorders (van Smeden et al., 2014; Kiezel-Tsugunova et al., 2018; Bhattacharya et al., 2019). The level of FASN, a key enzyme required for *de novo* fatty acid biosynthesis, increases rapidly following permeability barrier disruption and promotes epidermal homeostasis through several mechanisms (Ottey et al., 1995). FASN can enhance cell proliferative ability and maintain membrane synthesis (Vazquez-Martin et al., 2008). Furthermore, blocking the enzymatic activity of FASN can decrease phospholipid production, inhibit cell proliferation, and alter the metabolite profile in cancer cell lines (Jones and Infante, 2015). In addition, FASN is known to regulate and integrate with several signaling pathways, including the protein kinase C (PKC) and the PI3K/AKT/mTOR pathways, both of which are involved in cell growth and protein synthesis (Menendez, 2010; Benjamin et al., 2015). FASN inhibition can also hinder protein modifications by hampering palmitoylation, which is essential for skin barrier integrity (Ventura et al., 2015; Chen et al., 2020). Apart from the increased level of FASN, FASN expression was downregulated in *Krt17*-null mice following permeability barrier disruption. Our data indicated that K17 is a contributor to the epidermal barrier by regulating the expression of FASN, a key lipid synthesis enzyme, thereby affecting multiple processes related to FASN activity.

SREBP-1 is a key and well-characterized transcription factor for FASN. SREBP-1 is expressed as two isoforms, SREBP-1a and SREBP-1c. The SREBP-1a isoform was reported to be the main isoform expressed in human keratinocytes, where it plays a role in epidermal barrier function (Shimano and Sato, 2017). SREBP-1 has been demonstrated to function as an important node in the promotion of cell proliferation mediated by AMPK-dependent p70 ribosomal S6 kinase-1 (S6K1) (Düvel et al., 2010). Another study also highlighted the importance of SREBP activation in lipogenesis and cell development (Xu et al., 2020). More directly, liver X receptor (LXR), a SREBP-1 agonist, can stimulate lipid synthesis, lamellar body secretion, and post-secretory lipid processing, mechanisms that account for its ability to improve epidermal permeability barrier homeostasis (Man et al., 2006). It has also been shown that SREBP-1 upregulation results in increased expression of genes associated with lipid compounds and lamellar body formation in keratinocytes (Yokoyama et al., 2009). Independently of its transcriptional level, SREBP-1 proteolytic cleavage and subsequent translocation into the nucleus are required for its proper functioning (Eberlé et al., 2004). Mutants of sterol regulatory element-binding factor (*SREBF-1*), which encodes SREBP-1, display impaired cleavage and the absence of nuclear translocation. Genetic alterations in SREBP-1 also lead to the development of ichthyosis follicularis alopecia and photophobia (IFAP) syndrome, which is partially characterized by ichthyosis follicularis (Wang et al., 2020). These observations highlight the underlying influence of SREBP-1 in the lipid matrix of the permeability barrier and skin barrier functioning. As mentioned above, K17 has been reported to facilitate keratinocyte proliferation and protein synthesis through AKT/mTOR signaling (Kim et al., 2006). This pathway is a well-characterized modulator of SREBP-1 signaling through controlling the nuclear entry of lipin 1 (Peterson et al., 2011). Our data also verified the positive relationship between K17 expression and AKT/mTOR signaling activity (Supplementary Figure S2). Thus, it is likely that K17 triggers AKT/mTOR signaling and mediates SREBP promoter activity and its nuclear protein abundance. Here, we found that SREBP-1 cleavage and nuclear translocation were inhibited under K17 deficiency, which led to a delay in barrier repair. Parallel to the indirect modulation of SREBP-1 by AKT/mTOR signaling, we found that K17 interacts directly with flSREBP-1 *in vitro*. That K17 has been recently identified inside the nucleus of epithelial cells and was reported to exert direct effects on cell proliferation and gene expression suggests that K17 may be directly involved in SREBP-1 nuclear translocation (Depianto et al., 2010; Hobbs et al., 2016; Nair et al., 2021). The results of the present study provide further evidence that SREBP-1 signaling plays a crucial role in epidermal differentiation and skin barrier reordering and highlight the regulatory effect of K17 in this process. However, the precise molecular mechanisms underlining the direct interaction and signaling pathways between K17 and SREBP-1 require further exploration.

PPARs comprise a crucial set of transcription factors that control lipid metabolism, skin barrier permeability, inflammation, and cell proliferation and differentiation (Sertznig and Reichrath, 2011). Among the three PPAR isoforms, PPARγ is the main functional isoform in mammalian skin, especially during keratinocyte differentiation (Ramot et al., 2015). After acute disruption of the permeability barrier by either tape stripping or extraction of barrier lipids with repeated acetone treatment, recovery of permeability barrier function was shown to be accelerated in animals treated topically with PPARγ agonists (Man et al., 2006). One mechanism underlying this effect was reported to be that topical treatment with PPARγ activators increased cholesterol, fatty acid, and sphingolipid synthesis in the *epidermis* by inducing the mRNA expression of the corresponding enzymes (Schmuth et al., 2008). PPARγ activation also markedly stimulates the mRNA expression of ABCA12 in human keratinocytes in a dose- and time-dependent manner (Jiang et al., 2008). ABCA12, a member of the ABC superfamily of proteins, facilitates the delivery of sphingolipids to lamellar bodies in keratinocytes. Here, evidence obtained both *in vitro* and *in vivo* suggested that increased K17 expression resulted in a corresponding induction of PPARγ expression. Thus, we uncovered a significant role for K17 in promoting lipid metabolism through regulating the PPARγ signal and, perhaps, also maintaining the regular formation of a protective lipid matrix in the skin.

In conclusion, our data indicated that altered K17 levels may serve as an indicator of *epidermis* impairment. Our results further underlined that K17 plays an indispensable role in lipid metabolism in keratinocytes, as well as in the reconstruction of the skin barrier, *via* the modulation of SREBP-1 and PPARγ. Our findings suggest that the role of keratins may not be limited to structural support, but may also include the modulation of cellular metabolism. In addition, we propose that the upregulation of K17 serves primarily as a compensatory, protective response to barrier defects, thereby ensuring survival. However, the prominent proinflammatory effect resulting from an increase in K17 levels may also promote the deterioration of the epidermal defect, and in turn, lead to a further increase in K17 expression. Overall, our study provides a novel perspective regarding the role played by K17 in the pathology of skin diseases characterized by epidermal disruption.

## Data Availability

The original contributions presented in the study are included in the article/Supplementary Material, further inquiries can be directed to the corresponding authors.
