# Supplementary material for: Keratin 17 Is Required for Lipid Metabolism in Keratinocytes and Benefits Epidermal Permeability Barrier Homeostasis
Source: Front Cell Dev Biol. 2022 Jan 12;9:779257. doi: 10.3389/fcell.2021.779257 (PMC8790522; doi:10.3389/fcell.2021.779257)
Supplement: Supplementary file 1 [file DataSheet1.docx]

Supplementary Material

# Supplementary Figures and Tables

## Supplementary Figures


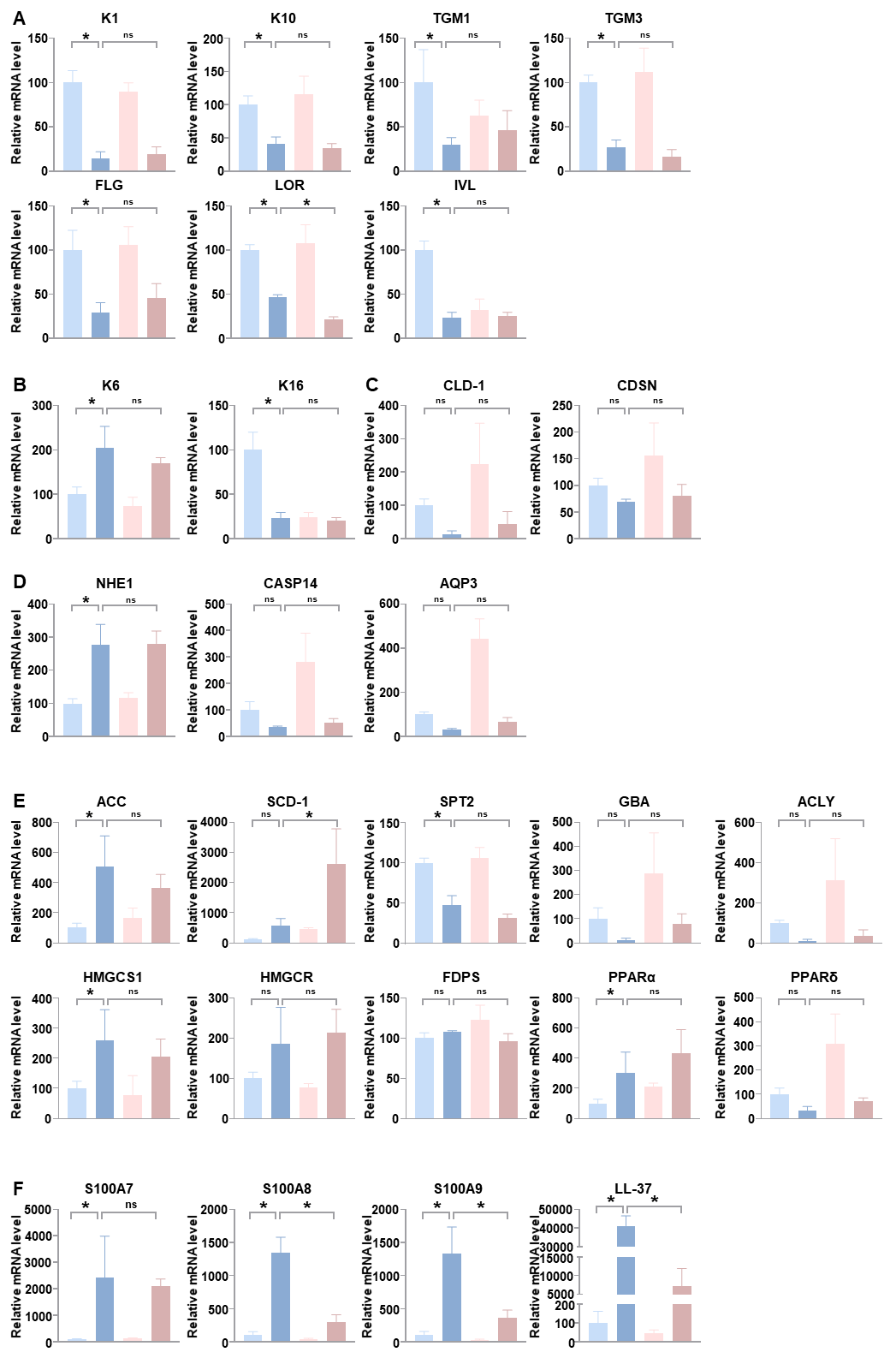


**Supplementary Figure 1.** Expression changes of genes indicating barrier function after acute epidermal disturbance. Quantitative real-time results showing mRNA expression levels of epidermal differentiation (A), proliferation (B), cell junctions (C), hydration (D), lipid metabolism (E) and antimicrobial peptides (F). Data were normalized to none-tape-stripped normal controls, setting controls as 100%. Data are representative for at least three independent experiments, and each group consisted of three mice. Results are shown as mean ± SEM. *P < 0.05


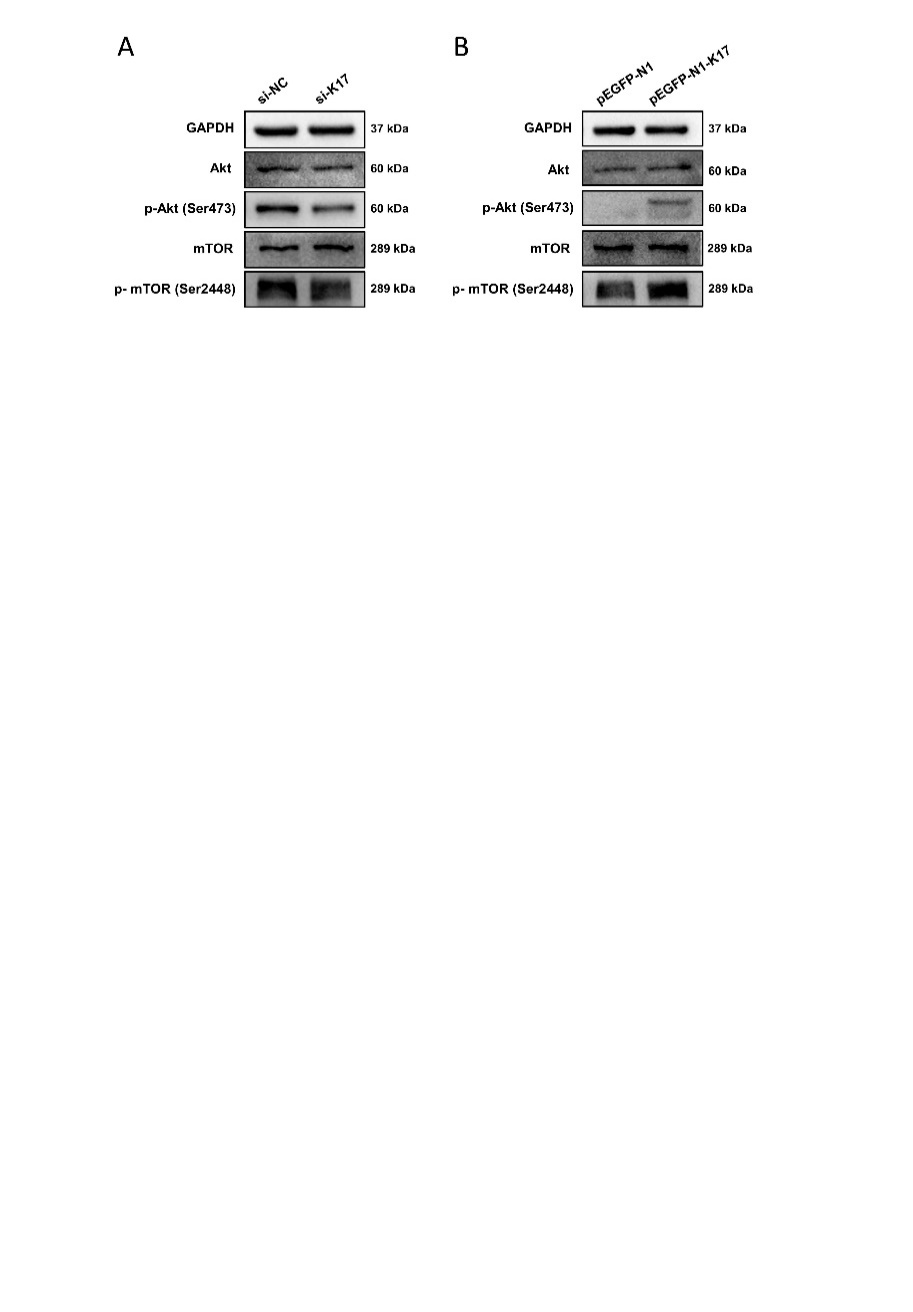


**Supplementary Figure 2.** Akt/mTOR activity regulated by K17 expression. (A) Protein levels of Akt, p- Akt (Ser473), mTOR and p- mTOR (Ser2448) were measured after transfection with K17 siRNA in keratinocyte cell line HaCaT cells. (B) Protein levels of Akt, p- Akt (Ser473), mTOR and p- mTOR (Ser2448) were measured after transfection with pEGFP-N1-K17 in HaCaT cells.

## Supplementary Tables

**Supplementary Table 1. siRNA sequences used in this study**

| Gene symbol | siRNA Sequences | |
| --- | --- | --- |
| Human *K17* siRNA | Sence | CCTGACTCAGTACAAGAAATT |
|  | Anti-sense | UUUCUUGUACUGAGUCAGGTT |
| Control siRNA | Sence | UUCUCCGAACGUGUCACGUTT |
|  | Anti-sense | ACGUGACACGUUCGGAGAATT |

**Supplementary Table 2. Primers for the analysis of mRNAs**

| Gene symbol | Sequence | |
| --- | --- | --- |
| Mouse *Actin* | Forward | CATTGCTGACAGGATGCAGAAGG |
|  | Reverse | TGCTGGAAGGTGGACAGTGAGG |
| Mouse *K17* | Forward | GCCCACCTGACTCAGTACAA |
|  | Reverse | GGAGCTGAGTCCTTAACGGG |
| Mouse *FASN* | Forward | CACAGTGCTCAAAGGACATGCC |
|  | Reverse | CACCAGGTGTAGTGCCTTCCTC |
| Mouse *PPARα* | Forward | ACCACTACGGAGTTCACGCATG |
|  | Reverse | GAATCTTGCAGCTCCGATCACAC |
| Mouse *PPARδ* | Forward | GGACCAGAACACACGCTTCCTT |
|  | Reverse | CCGACATTCCATGTTGAGGCTG |
| Mouse *PPARγ* | Forward | GTACTGTCGGTTTCAGAAGTGCC |
|  | Reverse | ATCTCCGCCAACAGCTTCTCCT |
| Mouse *ACC* | Forward | GTTCTGTTGGACAACGCCTTCAC |
|  | Reverse | GGAGTCACAGAAGCAGCCCATT |
| Mouse *SCD-1* | Forward | GCAAGCTCTACACCTGCCTCTT |
|  | Reverse | CGTGCCTTGTAAGTTCTGTGGC |
| Mouse *SPT2* | Forward | CCAGACTGTCAGGAGCAACCAT |
|  | Reverse | CTTCTTGTCCGAGGCTGACCAT |
| Mouse *GBA* | Forward | GCCAGTTGTGACTTCTCCATCC |
|  | Reverse | CGTGAGGACATCTTCAGGGCTT |
| Mouse *HMGCS1* | Forward | TGGTGGAAGCACAGTTGGCAAC |
|  | Reverse | CAGCATCATACACTCCTCAAAGC |
| Mouse *HMGCR* | Forward | GCTCGTCTACAGAAACTCCACG |
|  | Reverse | GCTTCAGCAGTGCTTTCTCCGT |
| Mouse *FDPS* | Forward | GGTGGTTCAGTGTCTGCTACGA |
|  | Reverse | CGCCTCATACAGTGCTTTCACC |
| Mouse *ACLY* | Forward | AGGAAGTGCCACCTCCAACAGT |
|  | Reverse | CGCTCATCACAGATGCTGGTCA |
| Mouse *K6* | Forward | GTGGCCTCAGCTCTTCTACC |
|  | Reverse | TCTGAGCACGGGATTCTGC |
| Mouse *K16* | Forward | TGGATGGCGAGAATATCCACAG |
|  | Reverse | GCTCCTTGAGGATGGACCG |
| Mouse *NHE1* | Forward | ACCTGTTCCTCACCGCCATCAT |
|  | Reverse | GTGTGGATCTCCTCGTTGATGG |
| Mouse *CASP14* | Forward | CTGAGAGGCAAGCCAAAGGTGT |
|  | Reverse | GAGCACAGCAACCTCATCTCCA |
| Mouse *AQP3* | Forward | CTCACCATCAACTTGGCTTTTG |
|  | Reverse | CATCGTAGTACAGCCCAAAAAC |
| Mouse *FLG* | Forward | GTCCCCTGACGGCTCC |
|  | Reverse | CTTGCTGCTGCACTTTGGAC |
| Mouse *LOR* | Forward | ACTCCTCTCAGCAGACCAGTCA |
|  | Reverse | AGAGGAGCCACCTCCACAGCT |
| Mouse *IVL* | Forward | AGGAGTCACCTGAGCCAGAACT |
|  | Reverse | TCAGGTGACTCCTGGTACTGCT |
| Mouse *K1* | Forward | ACAACCCGGACCCAAAACTT |
|  | Reverse | CTCTGCGTTGGTCCTCTTGT |
| Mouse *K10* | Forward | ACGAAGAGCTGGCCTACCTA |
|  | Reverse | CAGCAGCTGAGTCAGGTCAA |
| Mouse *TGM1* | Forward | ATCTGCCCTCAGGCTTTGATGG |
|  | Reverse | CGTTCTTGACGGACTCCACAGA |
| Mouse *TGM3* | Forward | ACATCAGCACCAAGGCAGTAGG |
|  | Reverse | CCTCGAAGATGTTGCGCCGAAA |
| Mouse *S100A7* | Forward | GATAGTGTGCCTCGCTTCATGG |
|  | Reverse | CTGGAGATGGTAGTCCTTCACC |
| Mouse *S100A8* | Forward | CAAGGAAATCACCATGCCCTCTA |
|  | Reverse | ACCATCGCAAGGAACTCCTCGA |
| Mouse *S100A9* | Forward | TGGTGGAAGCACAGTTGGCAAC |
|  | Reverse | CAGCATCATACACTCCTCAAAGC |
| Mouse *LL-37* | Forward | CTTCAACCAGCAGTCCCTAGAC |
|  | Reverse | GCCACATACAGTCTCCTTCACTC |
| Mouse *CLD-1* | Forward | GGACTGTGGATGTCCTGCGTTT |
|  | Reverse | GCCAATTACCATCAAGGCTCGG |
| Mouse *CDSN* | Forward | CCATCACCTCTGTCCAGAAACC |
|  | Reverse | TGTCCTTGGTGAAGTAGCCCAC |
| Human *β-Actin* | Forward | GGCTACAGCTTCACCACCAC |
|  | Reverse | TGCGCTCAGGAGGAGC |
| Human *K17* | Forward | CCAGCTCAGCATGAAAGCATC |
|  | Reverse | ACCTCTTCCACAATGGTACGC |
| Human *FASN* | Forward | TTCTACGGCTCCACGCTCTTCC |
|  | Reverse | GAAGAGTCTTCGTCAGCCAGGA |
| Human *PPARγ* | Forward | AGCCTGCGAAAGCCTTTTGGTG |
|  | Reverse | GGCTTCACATTCAGCAAACCTGG |
